# The food choice at work study: effectiveness of complex workplace dietary interventions on dietary behaviours and diet-related disease risk - study protocol for a clustered controlled trial

**DOI:** 10.1186/1745-6215-14-370

**Published:** 2013-11-06

**Authors:** Fiona Geaney, Jessica Scotto Di Marrazzo, Clare Kelly, Anthony P Fitzgerald, Janas M Harrington, Ann Kirby, Ken McKenzie, Birgit Greiner, Ivan J Perry

**Affiliations:** 1 Department of Epidemiology and Public Health, University College Cork, 4th Floor Western Gateway Building, Western Road, Cork City, Ireland; 2 School of Economics, University College Cork, Cork, Republic of Ireland; 3 School of Psychology, Trinity College Dublin, Dublin, Republic of Ireland

**Keywords:** Environmental modification, Nutrition education, Fat, Salt, Sugar, Workplace, Workplace complex dietary intervention

## Abstract

**Background:**

Dietary behaviour interventions have the potential to reduce diet-related disease. Ample opportunity exists to implement these interventions in the workplace. The overall aim is to assess the effectiveness and cost-effectiveness of complex dietary interventions focused on environmental dietary modification alone or in combination with nutrition education in large manufacturing workplace settings.

**Methods/design:**

A clustered controlled trial involving four large multinational manufacturing workplaces in Cork will be conducted. The complex intervention design has been developed using the Medical Research Council’s framework and the National Institute for Health and Clinical Excellence (NICE) guidelines and will be reported using the TREND statement for the transparent reporting of evaluations with non-randomized designs. It will draw on a soft paternalistic “nudge” theoretical perspective. Nutrition education will include three elements: group presentations, individual nutrition consultations and detailed nutrition information. Environmental dietary modification will consist of five elements: (a) restriction of fat, saturated fat, sugar and salt, (b) increase in fibre, fruit and vegetables, (c) price discounts for whole fresh fruit, (d) strategic positioning of healthier alternatives and (e) portion size control. No intervention will be offered in workplace A (control). Workplace B will receive nutrition education. Workplace C will receive nutrition education and environmental dietary modification. Workplace D will receive environmental dietary modification alone. A total of 448 participants aged 18 to 64 years will be selected randomly. All permanent, full-time employees, purchasing at least one main meal in the workplace daily, will be eligible. Changes in dietary behaviours, nutrition knowledge, health status with measurements obtained at baseline and at intervals of 3 to 4 months, 7 to 9 months and 13 to 16 months will be recorded. A process evaluation and cost-effectiveness economic evaluation will be undertaken.

**Discussion:**

A 'Food Choice at Work’ toolbox (concise teaching kit to replicate the intervention) will be developed to inform and guide future researchers, workplace stakeholders, policy makers and the food industry.

**Trial registration:**

Current Controlled Trials, ISRCTN35108237

## Background

Altering people’s health-related behaviours can have a substantial impact on the main causes of mortality and morbidity [1]. Behavioural interventions can modify current patterns of disease [1]. Diet-related disease is a major public health concern and continues to endanger our population health and the sustainability of our healthcare systems [2]. Dietary intake of fat (especially saturated fat and trans fat), sugar and salt play a critical role in the development of hypertension, obesity, type 2 diabetes and cardiovascular disease [3].

Dietary behaviour is influenced by a complex net of individual, environmental, societal, biological and psychological factors [4,5]. Given the complicated intricacies of dietary behaviour, there is a need to develop effective complex behavioural interventions to promote dietary change in the population. Complex interventions have several interacting components and should be developed systematically with appropriate evidence and theory [1,6]. These interventions should be piloted carefully and the process of implementation should be monitored [6].

The workplace is an ideal setting to implement these complex interventions, as most adults spend approximately 60% of their waking hours at work [7]. The workplace environment is a microcosm of society [8]. It is the most appropriate setting to examine complex dietary interventions as it can tolerate the interacting components of these interventions while assessing the impact in relatively homogenous workplace populations in controlled environments [8]. Relevant reviews agree that these interventions may be more effective if they are of high intensity, developed within a complex framework and comply with a robust study design [5,8-12].

However, there are substantial gaps in the current evidence base [8-13]. Although a moderate positive effect on dietary behaviour has been reported, particularly with fruit and vegetable intakes [9-12], workplace dietary intervention studies to date are of low-intensity with suboptimal study designs [9-13]. These interventions focus on information provision and fail to examine environmental approaches, such as food modification and real incentives, for example, price discounts [8]. Inconsistent reporting of previous studies has also precluded meta-analysis. Therefore, the impact of complex workplace dietary behaviour interventions is still unknown.

The aim of this study is to assess the effectiveness and cost-effectiveness of complex dietary interventions focused on environmental dietary modification alone or in combination with nutrition education in large manufacturing workplace settings. The study design is informed by the findings of a systematic review conducted by the authors [14].

This high-intensity complex intervention design is guided by appropriate structured frameworks and guidelines, including those of the Medical Research Council [6] and the National Institute for Health and Clinical Excellence (NICE) [1]. This study will be reported according to the TREND statement [6,15].

Environmental dietary modification and nutrition education approaches in this study will primarily draw on a soft paternalistic 'nudge’ theoretical perspective [16]. As recommended by the World Health Organization, catering and workplace stakeholders, including employees, will actively develop aspects of the intervention with the research team according to the specific characteristics of the included workplaces [4].

A process evaluation will be conducted. A cost-effectiveness economic evaluation will be undertaken in each workplace following a previous framework developed by Drummond *et al*. [17]. Very few studies have used cost-effective techniques to evaluate workplace interventions. Recently, Sacks *et al*. found that the traffic-light nutrition labelling offered excellent value for money as an obesity-prevention measure [18]. Absenteeism trends will also be monitored before and after the intervention to measure differences in labour productivity.

### Study hypothesis

Workplace complex dietary interventions that combine environmental dietary modification and nutrition education are more effective and cost-effective than nutrition education interventions alone or environmental dietary modification interventions alone when considering positive changes in dietary behaviour, health status and diet-related disease risk.

### Study objectives

The key objectives for this study are:

1. To develop long-term workplace complex dietary interventions focused on environmental dietary modification alone or in conjunction with nutrition education in large manufacturing workplace settings and evaluate the impact of these interventions on employees’ dietary behaviour, health status and nutrition knowledge.

2. To investigate employees’ food choice motives in a working environment.

3. To conduct a process evaluation that will include all key stakeholders to define critical elements in the success or failure of the complex dietary interventions.

4. To evaluate and compare the alternative interventions in terms of their costs and consequences.

## Methods/design

**Figure 1:**
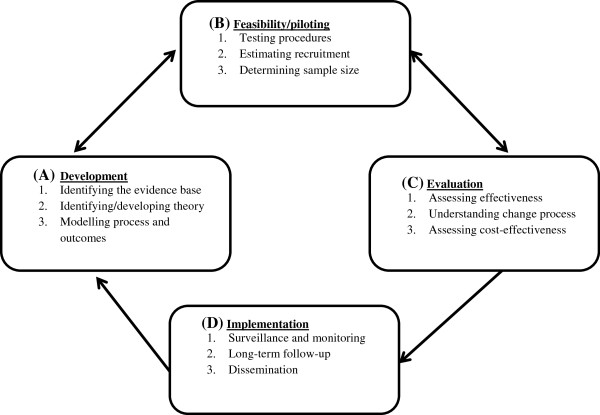
**Medical Research Council’s framework: 'Developing and evaluating complex interventions: new guidance’.** The complex intervention design has been developed and will be evaluated using this framework.

The complex intervention design has been developed and will be evaluated using the Medical Research Council’s framework [6]. The four phases of the framework are development, feasibility and piloting, evaluation, and implementation; these are illustrated in Figure 1.

### Development

This phase focuses on (1) identifying the evidence base, (2) identifying and developing a theoretical understanding, and (3) modelling the process and outcomes for the complex intervention.

#### Identifying the evidence base

We conducted a systematic review on the impact of workplace dietary modification interventions alone or in combination with nutrition education [14]. The review was guided by the PRISMA statement [19]. Although there was evidence that some interventions can moderately increase fruit and vegetable consumption, results show that uncertainty remains regarding the long-term effects on dietary behaviour, health status and economic cost. The systematic review findings informed the intervention design.

#### Identifying and developing theory

This intervention design will comply with a soft paternalistic 'nudge’ theoretical perspective [16]. The intervention will create positive reinforcement with indirect suggestions for healthy food choices to try to improve dietary behaviour with unforced compliance. Environmental engineering approaches will be guided by choice architecture that will include food modification, relocation of healthy food options and price discounts.

#### Modelling process and outcomes

**Figure 2:**
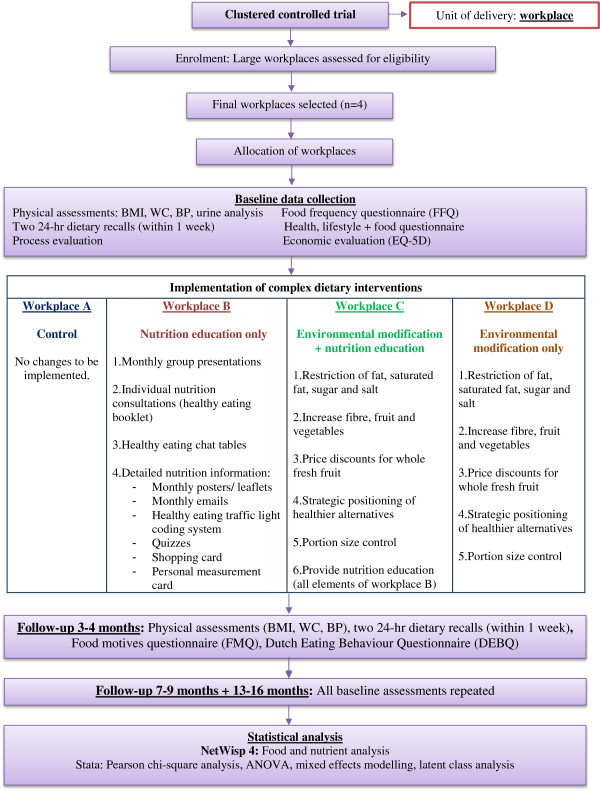
Food Choice at Work trial design.

This behavioural complex intervention design is guided by the detailed principles and recommendations of the NICE guidelines [1]. The study focuses on two potential methods to improve long-term dietary behaviour in the workplace including environmental dietary modification and nutrition education. Both methods will be measured independently and collectively in purposively selected workplaces. Workplace A (the control) will continue to follow usual practice. No changes will be implemented. Workplace B will receive nutrition education. Workplace C will receive nutrition education and environmental dietary modification. Workplace D will receive environmental dietary modification alone. The intervention design has been developed by the research team (nutritionists, dieticians, public health and health promotion researchers), catering stakeholders in Ireland (representatives of the Catering Managers Association of Ireland), workplace stakeholders (catering managers, human resources managers, occupational health managers) and the target population, that is manufacturing employees. Figure 2 illustrates the trial design.

Study outcomes will assess the effect of the intervention on dietary behaviours and improvements in diet-related disease risk. Primary outcomes will include changes in dietary behaviour and health status. Analysis of multiple 24-hour dietary recalls, food frequency questionnaires, food sales data and food purchasing patterns will indicate changes in dietary behaviour. Changes in body mass index (BMI), waist circumference, resting blood pressure and urinary electrolytes, including sodium and potassium (24-hr urine collections and random urine samples) will highlight improved health status outcomes. Secondary outcomes will determine food motives [20] and eating behaviours [21], changes in nutrition knowledge [22] and economic cost outcomes. A cost-effectiveness economic evaluation will be conducted and absenteeism trends will be recorded during the study period.

### Interventions

Each workplace will have a research workplace leader who will be based on-site for the duration of the study. The workplace leader will collaborate with the workplace stakeholders to co-ordinate the study and monitor daily adherence to the interventions.

#### Nutrition education in workplaces B and C

Nutrition education will include three components group presentations, individual nutrition consultations and detailed nutrition information.

Group presentations will consist of monthly 'lunch and learn’ group nutrition sessions (30 minutes per session) and will be delivered to all employees. These sessions will concentrate on portion control, reading food labels, general healthy eating, and reducing sugar, salt and fat dietary intakes. Sessions will be repeated a number of times per month so that all participants in all shifts have the opportunity to attend. Peer support and group discussion will allow for more effective learning.

Individual dietary counselling (20 minutes per session) with a nutritionist or dietician will be conducted with each participant at baseline, and follow-up sessions held at 3-4 months, 7-9 months and 13-16 months. The nutritionist or dietician will provide advice on how to follow a healthy diet, reach or maintain a healthy BMI and achieve or maintain a healthy resting blood pressure. The individual consultation will be based on the participant’s individual lifestyle, health status results (weight, BMI, waist circumference) and dietary recall assessments. The 'Food Choice at Work’ healthy eating booklet will be offered to each participant at the end of the first consultation. The booklet will support nutritional advice during consultations.

A 'healthy eating chat table’ will be situated outside the canteen during break times twice a month. All employees will have the opportunity to sit and ask a nutritionist or dietician about healthy eating.

Detailed nutrition information will be offered throughout the duration of the intervention using six key methods: (a) posters and leaflets, (b) emails, (c) menu labelling, (d) quizzes, (e) shopping cards, and (f) personalized measurement cards.

Posters and leaflets will be displayed throughout the workplace and based on the theme of the 'lunch and learn’ monthly nutrition sessions. This information will be replaced monthly.

Monthly emails will be disseminated to all employees using the workplace intranet to inform the employees of the scheduled activities for that month.

**Figure 3:**
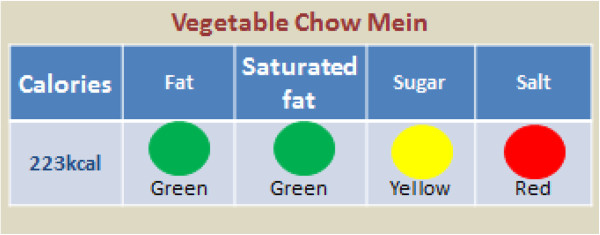
**Traffic-light display.** The traffic-light coding system will be applied to all food menus in workplaces that will implement the nutrition education intervention. The coding system will display the number of calories and traffic lights will show the amount of fat, saturated fat, total sugars and salt per portion size of the meal or food item. Traffic-light threshold values are guided by the Food Safety Authority of Ireland (FSAI) and the Food and Drug Administration labelling system.

A unique healthy eating traffic-light coding system will be applied to the daily menus in the employees’ canteens and vending machines on-site. The coding system will display the number of calories and traffic lights will show the amounts of fat, saturated fat, total sugars and salt per portion size of the meal or food item. The traffic lights will also be displayed in words for employees who are colour blind (Figure 3).

All traffic-light threshold values will be based on the Irish nutrient goals from the Food Safety Authority of Ireland (FSAI) and the Food and Drug Administration labelling system. The Irish nutrient goals have been developed on the basis of a caloric intake of 2000 kilocalorie (kcal) per day [23]. The recommended percentage intake is: for fat, 20% to 35% (<80 g); for saturated fat, <10% (≤20 g); for total sugar, ≤20% (≤90 g); and for salt, ≤6 g [23]. A green light will be applied if the food or meal does not exceed 5% of the recommended percentage intake. An amber light will be applied to a food or meal that contains between 5% and 20% of the recommended percentage intake. A red light will be applied if the food or meal exceeds the limit of 20% of the recommended percentage intake.

Two short quizzes focused on the traffic-light displays and the 'lunch and learn’ group nutrition sessions will be given to all employees each month. Randomly selected winners will receive free lunches.

Pocket-sized food choice shopping cards will be offered after baseline assessments. These cards will provide guidance on how to select healthy food choices when purchasing food at work or outside of work using our own unique traffic-light coding system.

Pocket-sized personal measurement cards (pocket size) will be offered after baseline assessments to allow participants to log and follow their progress throughout the study regarding their health status. Individual dietary advice from the nutrition consultations will also be recorded on the card.

#### Environmental dietary modification in workplaces C and D

The menus in workplaces C and D will be nutritionally analyzed using NetWISP software (Weighed Intake Software Program; Tinuviel Software, Warrington, UK) before the study commences. The workplace stakeholders and the research team will discuss and reach a consensus on all future environmental dietary modifications in the workplace canteens and vending machines. Taste testing will be conducted by the workplace stakeholders and the research team before the implementation of any modifications. All catering staff will be trained before and during the intervention period so there is high compliance with the specific dietary modifications and portion control.

The following five environmental dietary modifications will be recommended: (a) restriction of fat, saturated fat, sugar and salt, (b) increase in fibre, fruit and vegetables, (c) price discounts for whole fresh fruit, (d) strategic positioning of healthier alternatives and (e) portion size control.

For the restriction of fat, saturated fat, sugar and salt, all menus need to be modified. Stock and bouillon should be removed from all recipes and replaced with a recommended low-salt stock. Salt should be eliminated from all cooking processes. Fresh herbs, spices and garlic should be introduced to develop additional flavour. Savoury options that are high in salt, saturated fat and fat should be restricted (for example, sausage rolls, croissants) and replaced with low-fat or low-salt options. High-salt products (gravy mixes, stock cubes) and processed meats (bacon, corned beef) will be reduced and replaced where possible with low-salt options (turkey, chicken, fish). Fresh herbs, spices and garlic will be introduced to develop additional flavour. Ready-made meals will be removed and replaced with freshly cooked options. Full-fat dairy products (that is milk, cream, cheese and butter) will be replaced with low-fat options where possible. Cheese and cream will not be used as a garnish on meals. The amount of cheddar cheese will be reduced in all dishes. Cooking methods with oil, such as deep-fat frying, will be limited and will be replaced with methods of boiling, poaching, grilling, steaming and baking where possible. Only plant oils will be used in cooking (that is, rapeseed, olive, canola and other plant oils). Full-fat mayonnaise will be replaced with low-fat mayonnaise in sandwiches and other lunch options.

No sauces or accompaniments will be added to any meal unless the employee requests it. Chips and French fries will be removed from the menus two days a week and replaced with different potato options, for example baked potatoes. Pizzas will be removed from the menus three days a week. All desserts will be fruit-based. Soft carbonated drinks will be restricted and replaced with water, milk and unsweetened fruit juice options.

To increase fibre, fruit and vegetables, white pasta, rice and bread will be replaced with wholegrain alternatives. Fruit and vegetables will be added to rice, pasta, soup and meat dishes. A buffet-style fresh salad bar will be available to accompany any dish daily. Fresh whole fruit will be available throughout the day.

Portions of whole fruit will be offered at discount prices.

To introduce portion size control, workplaces will be recommended to comply with the FSAI guidance on portion size [23]. Training will be provided to all catering staff regarding strict portion size control. Standard serving tools will be used by caterers and employees to control portion size at mealtimes.

Healthier alternatives will be strategically positioned: healthy snacks, such as whole fresh fruit, dried fruit, nuts without chocolate, salt or sugar, brown sandwiches, brown soda bread and seeds will be positioned at eye level at the entrance of the canteen and in the vending machines. Chocolate, sweets, biscuits and crisps will be restricted and replaced where possible with healthy snacks in the canteen and in the vending machines located in the canteen. Full-size chocolate bars will be replaced with smaller options. Salt will be removed from the tables and will be replaced with sachets.

### Feasibility and piloting

The second phase includes (1) testing procedures, (2) estimating recruitment and (3) determining an appropriate sample size.

#### Testing procedures

In 2009, we carried out a cross-sectional comparison pilot study in two public hospitals in Cork, Ireland; one of which had implemented a long-term (2 years) catering intervention designed to reduce dietary fat, saturated fat, sugar and salt intake. All menus were modified. High-salt products (gravy mixes, stock cubes) and processed meat (bacon, corned beef) were replaced with low-salt options (turkey, chicken and fish). Fresh herbs, spices and garlic were introduced. Salt was removed in cooking. Saltcellars were removed from the tables in the canteen but small salt sachets were available at the service counter. Nutrition information was displayed in the canteen area. No sauces were added to any meals without the employee’s consent. All desserts were fruit-based. Staff members were encouraged to consume extra salad and vegetable options at no extra cost. Cooking methods with oil were reduced. No catering changes were implemented in the second hospital.

A total sample of 100 random employees aged 18 to 64 years (50 from each hospital) who consumed at least one main meal in the hospital staff canteen daily took part in the study. Dietary intakes and sociodemographic characteristics were assessed. Reported mean intakes of total sugars, total fat, saturated fat and salt were significantly lower in the intervention hospital when adjusting for age and sex. Estimated average salt intake in the intervention hospital (5.6 g/day) did not exceed the tolerable upper limit of 6 g/day vs. a mean salt intake of 6.7 g/day in the non-intervention hospital.

The study findings, published in the journal Public Health Nutrition [24], suggest that a structured catering initiative sustained over a relatively long period may influence long-term positive food choices at work and at home. Although these findings should be interpreted cautiously given the small sample size, many of the proposed dietary environmental modification and nutrition education components of the 'Food Choice at Work’ study have been shown to be acceptable and feasible in a workplace setting.

##### Validation study

A validation study to assess the accuracy of the 24-hour dietary recall method for calculating dietary salt intakes will be conducted after baseline data collection. CK will carry out a validation study to assess the accuracy of the study’s 24 hour dietary recalls for estimations of dietary salt intake in comparison with the 24-hour urinary sodium excretion method, spot urine samples and food frequency questionnaires.

#### Estimating recruitment

A four week period in each workplace will be allocated to estimate recruitment. The time taken to schedule employees and conduct baseline data collection appointments will be recorded to inform the other stages of data collection.

#### Determination of sample size

A decrease in BMI by 1 kg/m^2^ (1 unit) and a 2 g average fall in dietary salt intake would have population health significance and clinical significance in terms of the risk of diet-related disease, for example, hypertension. To detect this difference in BMI between the control and intervention groups at follow-up session after 7 to 9 and 13 to 16 months and assuming a common standard deviation of 3.77, it is estimated that a sample size of 448 (112 per workplace) would have 80% power at the 5% significance level (findings from a previous study show that a 1 kg/m^2^ difference was independently associated with 13% higher risk for hypertension) [25,26]. The study will also be adequately powered (80% power at the 5% significance level) to detect a fall in dietary salt intake between the control and intervention groups at follow-up periods of 7 to 9 and 13 to 16 months using a standard deviation of 4.2 (In a previous intervention study the response within each participant group was normally distributed with standard deviation 4.2) [27].

### Evaluation

The third phase is concerned with assessing the (1) effectiveness of the interventions, (2) understanding the change process and (3) assessing the cost-effectiveness of the complex intervention.

#### Effectiveness of the interventions

##### Study design

Effectiveness of the interventions will be evaluated using a clustered controlled trial design in four large manufacturing multinational workplaces based in Cork in the Republic of Ireland with a representative sample of employees.

##### Study duration

The total study duration is 16 months, with the interventions being delivered over a 9-month period. Follow-up is for 6 months post-intervention.

##### Unit of analysis

While the data will be collected at the individual level, the primary unit of analysis will be at the workplace level. Standard errors will be adjusted for clustering within the four workplaces before calculating confidence intervals.

##### Recruitment

A comprehensive list of manufacturing companies in Cork in the Republic of Ireland will be obtained from the Industrial Development Authority of Ireland (IDA) website. Workplaces will be systematically selected from the A to Z listing. As the focus of the study is to implement a complex dietary intervention in an ideal environment, workplaces will be purposively selected and allocated. Only workplaces and employees that meet the specified selection criteria will be recruited.

##### Inclusion criteria

Any manufacturing multinational workplace that employs more than 250 employees and has a daily workplace canteen for employees can be included in the study. The workplace must be located in Cork, represented on the Industrial Development Agency (IDA) website and able to commit to all components of the complex intervention for the duration of the study.

Any permanent, full-time employee who is contracted to work for the duration of the study period and purchases and consumes at least one meal in the main canteen daily will be eligible to participate in the study.

##### Exclusion criteria

All non-manufacturing national workplaces that employ less than 250 employees or do not have a workplace canteen; are not represented in the IDA website; not located in Cork or not able to commit to the intervention design for the study period will be excluded.

Employees will be excluded if they:

1. Work part time.

2. Do not have contracts to work during the study period.

3. Do not work in the workplace full-time (for example, work from home 2 days a week).

4. Travel regularly for work (more than once a month).

5. Do not purchase and consume a main meal from the staff canteen daily.

6. Are medically advised not to participate in the study.

7. Are involved in an on-going diet programme external to work (for example, the Weight Watchers programme).

##### Data collection methods

All data collection will take place during paid working hours (excluding employees’ breaks). To measure the effects on the primary and secondary outcomes, data will be collected in four stages using questionnaires, dietary and physical assessments and face-to-face semistructured interviews. Baseline assessments will be conducted prior to implementation of the intervention. Follow-up assessments will be carried out at 3 to 4 months, 7 to 9 months and 13 to 16 months.

##### Questionnaires

Four questionnaires will be self-completed by each participant electronically or in a hard-copy format. All questionnaires are based on validated, pre-tested questionnaires and will be completed at various study time-points.

The Health, Lifestyle and Food Questionnaire (HLFQ) is organized into ten different sections (A to J). Sections A, B, and C relate to the participant (sex, age, ethnicity, education), and include details of work life (permanent or temporary, job arrangement) and general health status (self-rated health, health conditions and self-rated weight) [27] Sections D, E and F relate to the participant’s usual dietary patterns at home and at work. Sections G, H and I investigate the participant’s usual lifestyle patterns including physical activity (using the International Physical Activity Questionnaire), smoking and alcohol questions [28]). Section J will focus on the participant’s nutrition knowledge using the General Nutrition Knowledge Questionnaire (GNKQ) [23]. The questionnaire will take approximately 25 minutes to complete.

The Food Motives Questionnaire (FMQ) will investigate motives underlying the selection of food [20]. It consists of nine scales, including health, mood, convenience, sensory appeal, natural content, price, weight control, familiarity and ethical concern. This questionnaire will help understand the causes of variation in dietary intake among participants. This questionnaire will take five minutes to complete.

The Dutch Eating Behaviour Questionnaire (DEBQ) is a validated eating behaviour scale to assess restrained, emotional and external eating behaviour [21]. The questionnaire will take three minutes to complete.

The EuroQol-5D (EQ-5D) is a standardized instrument for use as a measure of health outcome. Applicable to a wide range of health conditions and treatments, the EQ-5D health questionnaire provides a simple descriptive profile and a single index value for health status [29]. EQ-5D is primarily designed for self-completion by participants and is ideally suited for use in online surveys and face-to-face interviews.

##### Dietary assessments

The 24-hour dietary recall method will quantitatively measure current nutrient intake over a period of 24 hours, including the workplace and the home environment. Little burden is placed on the participant as this method requires short-term memory only but it fails to measure habitual diet. The Food Frequency Questionnaire tool is used to measure habitual dietary intake. It is a quantitative instrument and the most commonly used dietary assessment in large scale epidemiologic surveys.

The Food Frequency Questionnaire (FFQ) will be self-completed by each participant electronically or in a hard-copy format at baseline and follow-up at 7 to 9 months and 13 to 16 months. The FFQ is an adapted version of the European Prospective Investigation of Cancer FFQ [30]. It has been used extensively in the Irish population including the Irish Surveys of Lifestyle, Attitudes and Nutrition [28] and the original Cork and Kerry baseline study in 1998 [31] and the baseline Phase II Cork and Kerry study in 2010 [32]. The FFQ is designed to assess the whole diet and includes 150 food items arranged into the main food groups. Respondents will be asked to record their average frequency of consumption of each food item over the last year. Typical weights, portion sizes and nutrient intake will be based on recommendations established by the Food Standards Agency (2002) [33] and McCance and Widdowson’s Food Composition Tables [34,35]. A specifically designed programme, NetWISP4© (Weighed Intake Software Program; Tinuviel Software, Warrington, UK), will convert dietary information to food quantities and nutrient values [36,37].

The 24-hour dietary recall method will be a modified version of the validated UK 24 hour dietary recall method [38]. Two dietary recalls will be collected within one week to examine on- and off-work-duty dietary patterns at baseline, and follow-up periods of 3 to 4 months, 7 to 9 months and 13 to 16 months.

The three-step method will outline specifically what the participant had to eat and drink in the previous 24 hour period.

1. Quick list: participants will be asked to report everything that they had to eat or drink the day before their appointment (midnight to midnight).

2. The nutritionist or research assistant will collect detailed information on items named in the quick list (consumption time, place of consumption, brand and recipe), foods likely to be eaten in combination (milk in coffee) and the quantity consumed and any leftovers or second helpings.

3. Recall review: participants will have an opportunity to provide additional information or to refer to foods forgotten in the quick list.

Finally, the interviewer will ask the participants about their consumption of water and food supplements. All information gathered is recorded in a food consumption record. Additional modifications added to the method include specific prompts to measure salt and oil consumption. Each 24-hour dietary recall data collection will take approximately 20 minutes to complete. Finally, the nutritionist or research assistant will complete an interviewer evaluation. Each food, drink and portion size will be coded according to the 24-hour coding instructions based on the validated UK method. Food and nutrient analysis will be calculated using NetWISP4© (Weighed Intake Software Program; Tinuviel Software, Warrington, UK) [36,37].

##### Physical assessments

Each participant will be asked to participate in a physical assessment where BMI, (midway) waist circumference, waist hip ratio and resting blood pressure will be measured.

##### Resting blood pressure

Blood pressure measurements will be obtained using the Omron M7 Digital Blood Pressure monitor. It is a compact, fully automatic monitor, operating on the oscillometric principle. This method of measurement determines the participant’s blood pressure by measuring the pressure fluctuations caused by the pulse waves. Before the measurement begins, the participant will be seated and as relaxed as possible with both feet parallel and flat on the floor. The researcher will ensure that the participant has not been smoking or participating in any vigorous exercise prior to the measurement. A full bladder also affects a blood pressure reading, so the researcher will give the participant an opportunity to void prior to measurement.

The researcher will instruct the participant to remove any tight clothing covering the upper arms and ensure that the participant has been seated and settled for approximately 5 minutes prior to commencing the procedure. The measurements will be taken on the right arm whenever possible. The participant’s arm will rest on a desk so that the antecubital fossa (a triangular cavity of the elbow joint that contains a tendon of the biceps, the median nerve and the brachial artery) is at the level of the heart and the palm is facing up. The participant must always feel comfortable. The greatest circumference of the upper arm will be measured for a suitable cuff, with the arm relaxed and in the normal blood pressure measurement position (antecubital fossa at the level of the heart), using an inelastic tape. Three measurements will be taken from each participant one minute apart.

##### Urine analysis

Spot urine samples will be obtained for analyses of sodium, potassium, urea and creatinine levels. Two spot urine samples will be obtained from each individual at baseline and follow-up periods of 7 to 9 and 13 to 16 months (six spot samples in total per participant). Each participant will provide one sample from the evening before their on-duty 24-hour dietary recall and their second sample will be the first sample voided on the morning of their dietary recall. The urine samples will be taken approximately 12 hours apart, for example, 8 pm and 8 am.

A subsample of participants from each workplace will be asked to complete a 24-hour urine collection the day before their on-duty 24-hour dietary recall at baseline and at follow-up periods of 7 to 9 and 13 to 16 months. 24-hour urinary sodium excretion is considered the gold standard method for estimating dietary salt intake. It is estimated that between 90% and 95% of dietary salt intake is excreted in urine. Para-aminobenzoic acid, a biologically inert substance that is rapidly excreted in urine, will be administered to all participants on the day of urine collection to validate the completeness of the 24-hour collection sample. To estimate total sodium excretion in the spot urine samples, the sodium content will corrected for total 24-hour urine volumes calculated from the validated 24-hour urine samples collected.

##### Statistical analysis

Data will be recorded manually and entered electronically into a Stata statistical software package prior to statistical analysis. Data manipulation and statistical analyses will be conducted using Stata. Primary analysis will examine the effects of the interventions by measuring changes in dietary behaviour, health status outcomes and nutrition knowledge.

Data regarding individual and environmental factors that may influence the effectiveness of the dietary complex interventions will be collected during baseline and follow-up. Individual factors will include personal (age, sex, ethnicity, education status, nutrition knowledge), lifestyle (smoking status, alcohol consumption, physical activity) and workplace factors (shift-work patterns, work status, for example, production worker, work schedule). Environmental factors will include the employees (sex breakdown and age profile) and the workplace structure (number of employees in workplace, canteen arrangement, for example, opening hours, employee structure, for example, percentage of employees working in production).

Proportions in workplaces A, B, C and D will be compared using Pearson’s chi-square and McNemar’s test for paired data. Mean levels of macronutrients, fibre, salt, fruit and vegetable intake in workplaces A, B, C and D will be compared and analyzed using a repeated measure analysis of variance (ANOVA) model. To measure nutrition knowledge, all participant responses to the GNKQ will be coded numerically and converted to a corrected score, as defined by Parmenter and Wardle [22].

A mixed effects model will examine subject variation in the longitudinal trends in dietary behaviour, explore associations between trends in dietary behaviour and health status over time in workplaces A, B, C and D and adjust for the potential confounding effect of other factors such as age, sex and shift-work patterns. The cost-effectiveness economic evaluation will be completed using a similar framework to Drummond *et al*. [17] and Roberts *et al*. [39].

##### Planned subgroup analysis

Secondary analysis will investigate external factors that may be associated with the effects of the interventions. Subgroup analysis will look for possible differential effects in different employee disciplines (that is, production employees versus management) and work groups that is, shift workers versus day workers). Analysis will be conducted across workgroup-strata and education level as a proxy measure of social class. Dietary pattern analysis will be conducted using latent class analysis [40]. It will identify mutually exclusive subgroups within different dietary classes. Latent class analysis will estimate each participant’s probability of belonging to a particular dietary class. A change in these subject-level probabilities is evidence of changes in dietary behaviour and preference. Changes in dietary preferences will be compared in all workplaces and associations with clinical and behavioural outcomes will be examined.

#### Understanding change process: process evaluation

The implementation of the intervention will be monitored with a detailed process evaluation throughout the intervention period. A subsample of key workplace stakeholders from each workplace will be involved in a process evaluation to define critical elements in the success or failure of the complex dietary interventions through the use of semistructured interviews. Workplace stakeholders (catering managers, human resources managers, occupational health managers and employee representatives) will include individuals who have been exposed to the intervention either by participation or have been involved in the development of the study design.

Semistructured interviews will be conducted with participants for one hour at baseline and follow-up after 7 to 9 and 13 to 16 months. Further to this, the researchers tasked with implementing the study will also be involved in the on-going process evaluation. They will participate in focus groups and document study activities on a weekly basis.

The process evaluation will explore opinions on effective strategies to promote healthy eating at work, determine participants’ perceptions of the implementation of the interventions in their workplace settings and examine the workplace stakeholders’ awareness of changes in the workplace and changes in their dietary patterns for the duration of the intervention.

The process evaluation plan will be directed by Steckler and Linnan’s conceptual framework [41]. The topic guide will be based on the following six components: fidelity, dose delivered, dose received, reach, recruitment and context. With informed participant consent, the interviews and focus groups will be digitally recorded, transcribed and analyzed using NVIVO software (QSR International Pty Ltd). A framework approach will be used for data analysis [42]. This method is appropriate given that the study has pre-specified objectives but it will also allow for unexpected themes to emerge [43].

#### Assessing cost-effectiveness: economic evaluation

A framework similar to that described in Drummond *et al*. [17] and Roberts *et al*. [39] will be used to measure the cost-effectiveness of each intervention. Seven steps will be followed:

1. Describe each program alternative, its components and potential benefits.

2. State the perspective from which the programmes will be analyzed. The principal costs of the interventions are the advice by the nutritionists and the toolbox (resources used for implementation of interventions: training, equipment). If these costs are borne by the businesses, then the perspective will be that of the business and their staff (the business benefits from lower sick days, the staff from better health). If the health service bears these costs, then the perspective is that of the health service (it bears the costs, but sees an improvement in population health, which is the primary objective of the health service). Thus the perspective adopted will depend on who is bearing the costs and reaping the benefits.

3. Identify, measure and value the costs of the alternatives. The main costs will be the toolbox, the on-going advice of nutritionists to construct a healthy cost-neutral menu and the printing costs for provision of information. Identification involves the listing of all resources used; measurement captures the resources used in physical units and valuation puts prices on these physical resources. We will also measure sick days for each employee the year before the intervention and the year after the intervention and compare the two results to measure whether there is a difference.

4. Identify, measure and value the outcomes of the alternatives. The primary outcome will be quality of life as measured using EQ-5D. A secondary outcome will be BMI. Some of the health status outcomes data are already collected in Work Stream 1 with questions on BMI and waist circumference included.

5. Future costs and outcomes will be discounted at the appropriate discount rate. In Ireland this is taken at 3.5% and in the UK it is 5%.

6. Decision analytical modelling will be used to assess parameter uncertainty and heterogeneity. For instance, quality-adjusted life years will be calculated based on a combination of the quality of life scores emerging from the EQ-5D measurement and the number of life years saved, based on extrapolation of the changes in BMI. The uncertainty surrounding these estimates will be appropriately modelled.

7. Incremental cost-effectiveness ratios will be calculated for each of the alternatives and analysis of relative value for money will be reported. This and other measures of value for money, such as net benefit, will be presented in a decision analytical framework.

### Implementation

The fourth phase concentrates on (1) surveillance and monitoring, (2) long-term follow-up and (3) dissemination.

#### Surveillance and monitoring

As the workplace leaders will be based in the workplace during the study period, they will observe and enforce all components of the intervention and record a weekly log of the intervention activities. The workplace leaders will meet with the workplace stakeholders on a weekly basis. The workplace leaders will inform the 'Food Choice at Work’ logistics committee.

The 'Food Choice at Work’ logistics committee will meet monthly in each workplace to monitor the efficiency of day-to-day data collection, harmonize communication, discuss concerns relating to the study design and data, discuss training of the research team and participant or stakeholder safety. Members will include the project manager, lead investigator (FG), workplace leader, human resources representative, occupational health and safety manager, employee representative and catering manager from each workplace.

The steering and data monitoring committee will meet once every two months. Members will include the lead investigator, principal investigator, co-investigators (with expertise in nutritional science, behavioural science, health economics, epidemiology, public health and biostatistics), the project manager and workplace leaders. The committee will monitor the study; oversee day-to-day ethical, data and administrative management; monitor compliance with the intervention and discuss dissemination. Quarterly progress reports relating to budget forecasts and fieldwork progress will be made.

An oversight committee will meet quarterly to review study deliverables and outputs, ensure that accurate, timely and appropriate reporting and problem solving occurs. Financial management will also be discussed. Members will include the principal investigator (IJP), lead investigator (FG), project manager, representative from the office of research and innovation and the finance department in the University of College, Cork, Republic of Ireland.

#### Long-term follow-up

The complex interventions will be implemented over a 9-month period and long-term follow-up will take place at 3 to 4 months, 7 to 9 months and 13 to 16 months (6 months post-intervention). We feel that 16 months is necessary to measure the sustainability of changes in dietary behaviour but if additional funding is available, we would consider a further follow-up phase at 24 months.

#### Dissemination

Future academic dissemination will occur through a range of academic international peer reviewed journals. National and international conferences will be attended to disseminate research findings using posters and oral presentations. Employees in the included studies will be informed by email. We will use modern social media technology, including a 'Food Choice at Work’ Facebook page and regular tweets from our study Twitter account to inform the general public. Noteworthy findings will be published in future press release to inform the public, food industry and public health policy makers. A 'Food Choice at Work’ toolbox (concise teaching kit to replicate the intervention) will be developed to inform and guide future researchers, relevant stakeholders and policy makers.

### Threats to validity

#### Selection bias

Selection bias will be minimized in this study. All employees will be masked to the study hypothesis. Employees will be informed of a university-led study observing employees’ general dietary intakes over a 13 to 16 month period. No additional information will be provided to employees about the study design. Participants will be randomly selected using random number allocation software.

#### Information bias

This study is open to information bias including recall bias and interviewer bias.

##### Recall bias

It is not feasible to adequately blind study participants to the changes in their workplace environments, for example traffic-light labelling, therefore recall bias may be an issue. Participants will be interviewed by the research team in a standardized manner. The validated questionnaires are designed to measure potential co-founders and co-factors associated with the effectiveness of these interventions. Outcomes will be measured objectively where possible. Changes in health status will be measured using BMI, waist circumference, resting blood pressure and urinary electrolytes. Economic cost will be monitored using absenteeism trends and changes in profit margins in the workplace canteens will be recorded.

##### Interviewer bias

It is not possible to adequately blind the research team to the allocation of workplaces. Therefore, it is possible that the research team may act differently depending on the workplace allocation. To minimize this bias, the research team will be adequately trained and retrained at specific time-points to comply with a cohesive protocol to ensure all data is collected in a standardized manner. Data will be continuously monitored to ensure that research members are not inviting a systemic error (bias) into the data.

### Side-effect reporting and quantification

Reporting will adhere to the TREND guidelines [6,15]. No adverse events are envisaged for participants. The field work will be carried out in compliance with a detailed standard operating procedures manual. All field research employees will receive formal training for dietary and physical assessments at baseline and retraining before follow-up periods to ensure standardization of processes and procedures. All scales, tape measure and automated blood pressure monitors will be calibrated and recorded at the start of the study and recalibrated monthly in accordance with the manual.

Urine samples (24 hour urine collections and spot urine samples) will be assayed for electrolytes in an accredited hospital laboratory. The manual explains in specific detail the standard duty of care for abnormal blood pressure and urine results. The first priority will always be the health and wellbeing of the participant. A physician working with the Department of Epidemiology and Public Health, University College Cork, Republic of Ireland will oversee all 24 hour urine collection results and advise accordingly.

## Discussion

The 'Food Choice at Work’ study is the first high-intensity, complex dietary intervention study to measure the effectiveness and cost-effectiveness of environmental modification and nutrition education over a long-term period in similarly structured controlled manufacturing workplaces. This unique study will be developed and evaluated according to an established academically rigorous framework and has the potential to improve dietary behaviour, nutrition knowledge and reduce the risk of diet-related disease.

### Strengths

The study is developed using systematic theory and an evidence base. It is developed and evaluated according to the TREND statement (an academic framework recommended by the Medical Research Council’s and NICE guidelines [6,15]).

The study has a participatory approach, and includes catering and workplace stakeholders in the study design and evaluation. It has a complex 'high-intensity’ intervention design including a unique traffic-light coding system based on recommended portion size. Intensive training will be provided for the research team and caterers (workplaces C and D).

There is no risk of contamination, as all employees work in different companies located in different geographical areas.

There is a triangulation of methods. The dietary, health status and knowledge assessments will provide descriptive and contextual data on changes due to the intervention, while the semistructured interviews will deepen our understanding of the process of the implementation according to the perspectives of key stakeholders within the intervention workplace.

Various outcome measures will be used to assess changes in dietary behaviour and health status (objective and self-reported measures). Objective measurements include BMI, resting blood pressure and urine analysis (24-hour urine collection and spot urine samples). Self-reported measures include the completion of questionnaires (HLFQ, FFQ, FMQ, DEBQ and EQ-5D).

The study will have a thorough process evaluation and extensive cost-effectiveness economic evaluation. Study progress will be monitored by the logistics committee in all workplaces and the steering committee.

### Limitations

The study design is not randomized. However, the characteristics of each workplace are similar, including work schedules (shift patterns), company type (production and office based), skilled and educated workforces. Demographic information from the questionnaires will determine further comparison between worksites. The sample will also be randomly selected from the employee lists.

There is no concealment of allocation. The workplaces were purposively selected to ensure that all components of the interventions could be implemented successfully.

There is a lack of blinding: given the nature of the workplace interventions (nutrition consultations or environmental change), it is not possible to adequately blind personnel or participants. Participants will be masked to the study hypothesis.

There is a selection bias: healthy employees may be more likely to participate but demographic variables of non-responders will be examined to ensure the participants are representative of the general workforce.

Recall bias and social desirability bias may be evident, given that both dietary measurements (FFQ and 24-hour dietary recall) are self-reported. Dietary data may be overestimated or underestimated. The FFQ will be completed by the participant without the presence of the researcher. The 24-hour dietary recall method is clearly structured with specific food prompts so recall bias may be prevented. The researcher will start each visit with the 24-hour dietary recall and will not comment about food before the recall so social desirability bias may be reduced.

### Implications for research and practice

The Food Choice at Work interventions may improve the included employees’ dietary behaviours and reduce their diet-related disease risks. This study will provide critical evidence of the effectiveness of workplace complex interventions in the promotion of healthy dietary behaviours in the manufacturing working population. It may assist in the development of future guidelines to improve dietary behaviours in the workplace and will inform future researchers. It may influence national and international catering stakeholders, policy makers and motivate the food industry to provide healthier food choices. If the findings are positive, it may reduce diet-related disease development and the burden on the healthcare system in the Republic of Ireland. The large multinational manufacturing companies included in the study have similar worldwide structures and operations; the findings, therefore, will be generalizable nationally and transferable internationally. Following study completion, it is planned to examine the transferability of this complex intervention in a European or global context.

### Ethics

Ethical approval was granted by the Clinical Research Ethics Committee of the Cork Teaching Hospitals in the Republic of Ireland in May 2012 and was amended in March 2013. Permission has been granted by the managing directors and catering managers in all workplaces. Informed consent will be obtained from all participants prior to participation in the study.

## Trial status

Recruitment of participants is on-going (July 2013). Data collection will not be completed until at least December 2014. Trial registration: Current Controlled Trials ISRCTN35108237.

## Abbreviations

ANOVA: Analysis of variance; BMI: Body mass index; DEBQ: Dutch Eating Behaviour Questionnaire; EQ-5D: EuroQol-5D; FFQ: Food Frequency Questionnaire; FMQ: Food Motives Questionnaire; FSAI: Food Safety Authority of Ireland; GNKQ: General Nutrition Knowledge Questionnaire; HLFQ: Health, Lifestyle and Food Questionnaire; IDA: Industrial Development Authority of Ireland; kcal: Kilocalorie; NICE: National Institute for Health and Clinical Excellence.

## Competing interests

The authors declare that they have no competing interests.

## Authors’ contributions

FG was primarily responsible for the final content of the paper and is the guarantor. FG, JSDM, BG, KMcK and IJP worked on the study design. AK designed the methods for the cost-effectiveness economic evaluation. JMH, IJP, JSDM, CK and FG were involved in developing the data collection methods. APF and FG were responsible for the sampling methods and data analysis plan. FG and IJP co-wrote the paper. All authors approved the final version of the paper for publication.
